# Chitosan bead containing metal–organic framework encapsulated heteropolyacid as an efficient catalyst for cascade condensation reaction

**DOI:** 10.1038/s41598-023-29548-2

**Published:** 2023-02-16

**Authors:** Samahe Sadjadi, Neda Abedian-Dehaghani, Abolfazl Heydari, Majid M. Heravi

**Affiliations:** 1 Gas Conversion Department, Faculty of Petrochemicals, Iran Polymer and Petrochemical Institute, P.O. Box 14975-112, Tehran, Iran; 2 Department of Chemistry, School of Physics and Chemistry, Alzahra University, P.O. Box 1993891176, Vanak, Tehran, Iran; 3 Polymer Institute of the Slovak Academy of Sciences, Dúbravská Cesta 9, 845 41 Bratislava, Slovakia

**Keywords:** Chemistry, Catalysis, Heterogeneous catalysis

## Abstract

Using cyclodextrin and chitosan that are bio-based compounds, a novel bi-functional catalytic composite is designed, in which metal–organic framework encapsulated phosphomolybdic acid was incorporated in a dual chitosan-cyclodextrin nanosponge bead. The composite was characterized via XRD, TGA, ICP, BET, NH_3_-TPD, FTIR, FE-SEM/EDS, elemental mapping analysis and its catalytic activity was examined in alcohol oxidation and cascade alcohol oxidation–Knoevenagel condensation reaction. It was found that the designed catalyst that possess both acidic feature and redox potential could promote both reactions in aqueous media at 55 °C and various substrates with different electronic features could tolerate the aforementioned reactions to furnish the products in 75–95% yield. Furthermore, the catalyst could be readily recovered and recycled for five runs with slight loss of the catalytic activity. Notably, in this composite the synergism between the components led to high catalytic activity, which was superior to each component. In fact, the amino groups on the chitosan served as catalysts, while cyclodextrin nanosponge mainly acted as a phase transfer agent. Moreover, measurement of phosphomolybdic acid leaching showed that its incorporation in metal–organic framework and bead structure could suppress its leaching, which is considered a drawback for this compound. Other merits of this bi-functional catalyst were its simplicity, use of bio-based compounds and true catalysis, which was proved via hot filtration.

## Introduction

In recent decades, unprecedented rate of technological growth resulted in increase of environmental concerns^1^. As a solution, development of environmentally benign and practical synthetic methods that meet the criteria of green chemistry is on the agenda. In this regard, cascade reactions^2^ that combine consecutive synthetic steps in one-pot and benefit from advantages, such as less energy consumption, less waste production and avoiding intermediate separation and purification are great tools for developing sustainable chemistry. To promote cascade reactions, multifunctional catalysts are required^3^, in which several catalytic active sites are introduced on a recyclable supporting material. In this context, wise choice of the support and immobilization strategy can provide synergistic effects and furnish catalysts with high efficiency. One of the well-known cascade reactions is alcohol oxidation-Knoevenagel condensation reaction that is used for the synthesis of α,β-unsaturated nitriles. The key step in this cascade reaction is selective oxidation of alcohols to benzaldehydes, which can be catalyzed by various catalysts, such as enzymes^4^, metal oxides^5^ and heteropolyacids that are non-corrosive and non-toxic inorganic metal oxide clusters^6^ with redox potential and acidic properties. Heteropolyacids can catalyze various chemical transformations^7–11^, ranging from epoxidation to photo-degradations^12–15^. The main shortcoming of this class of inorganic clusters is their high solubility in conventional solvents that resulted in their onerous and inefficient recovery and recyclability^13^. As a solution to this problem, heteropolyacids have been stabilized on supporting compounds to furnish heterogeneous catalysts^16^. Although this strategy is helpful, leaching of heteropolyacids from the support is still a challenging issue. Knoevenagel condensation, on the other hand, can be promoted by various catalysts^17–19^, such as metal–organic frameworks (MOFs) and chitosan (CS)^20–22^. MOFs are porous nanomaterials that are composed of metallic centers^23–27^ and organic linkers^28–30^ and mostly possess Lewis acidic sites. In fact, high dispersion of metal ions in the MOF frameworks allows them to interact with reagents. Other features of MOFs that make them interesting candidates for catalysis are their porous nature^31^ and the possibility of tuning their properties through altering the linkers and metal centers^32^. To date various MOFs have been developed and utilized for various applications, including catalysis^33–37^. One of the mostly used MOFs is MIL-101(Fe), which is prepared from iron precursors and terephthalic acid and widely used for catalytic purposes^38^. Despite all merits of MOFs, their relatively time-consuming synthetic procedure as well as the necessity of use of toxic solvents and costly starting materials limit their large scale uses and render MOF-based catalytic systems relatively expensive. Hence, use of MOF in combination with other components to form composites can be considered as a solution^39^.

CS is an abundant carbohydrate^40^ that benefits from myriad Brønsted acidic sites and Lewis basic sites in its backbone^41^. This carbohydrate has been extensively utilized for catalysis and to date various chemical transformations have been promoted via CS, CS-based composites and CS-beads^42–45^. Recently, it has been proved that CS-coated MOF could be a potential catalytic system for deacetalization-Knoevenagel cascade reaction^41^. Another approach for developing green and environmentally friendly methodologies, is omitting toxic solvents and promoting reactions in aqueous media that offers many economic advantages. Unfortunately, for the chemical transformations, in which hydrophobic substrates are used, immiscibility of reagents in aqueous media may cause low conversion and yield. To circumvent this issue, use of phase transfer agents, such as cyclodextrins (CDs) that are cyclic oligosaccharides with hydrophilic outer surfaces and hydrophobic cavities is suggested^46–48^. CDs can encapsulate the hydrophobic substrates inside their cavities and transfer them into aqueous media. Furthermore, CD-based polymeric systems, such as CD nanosponge (CDNS), are also applied for this purpose^49,50^. In fact, CDNSs are 3-D polymeric networks, achieved from cross-linking of CD with proper cross-linking agents, such as dimethyl carbonate and diphenyl carbonate. Compared to CD, CDNS benefits from heterogeneous nature, multiple CD units, various pores with different sizes, which allow encapsulation of various substrates^51,52^.

**Scheme 1:**
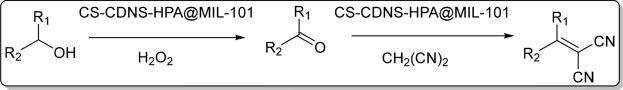
Cascade alcohol oxidation–Knoevenagel condensation reaction.

**Figure 1:**
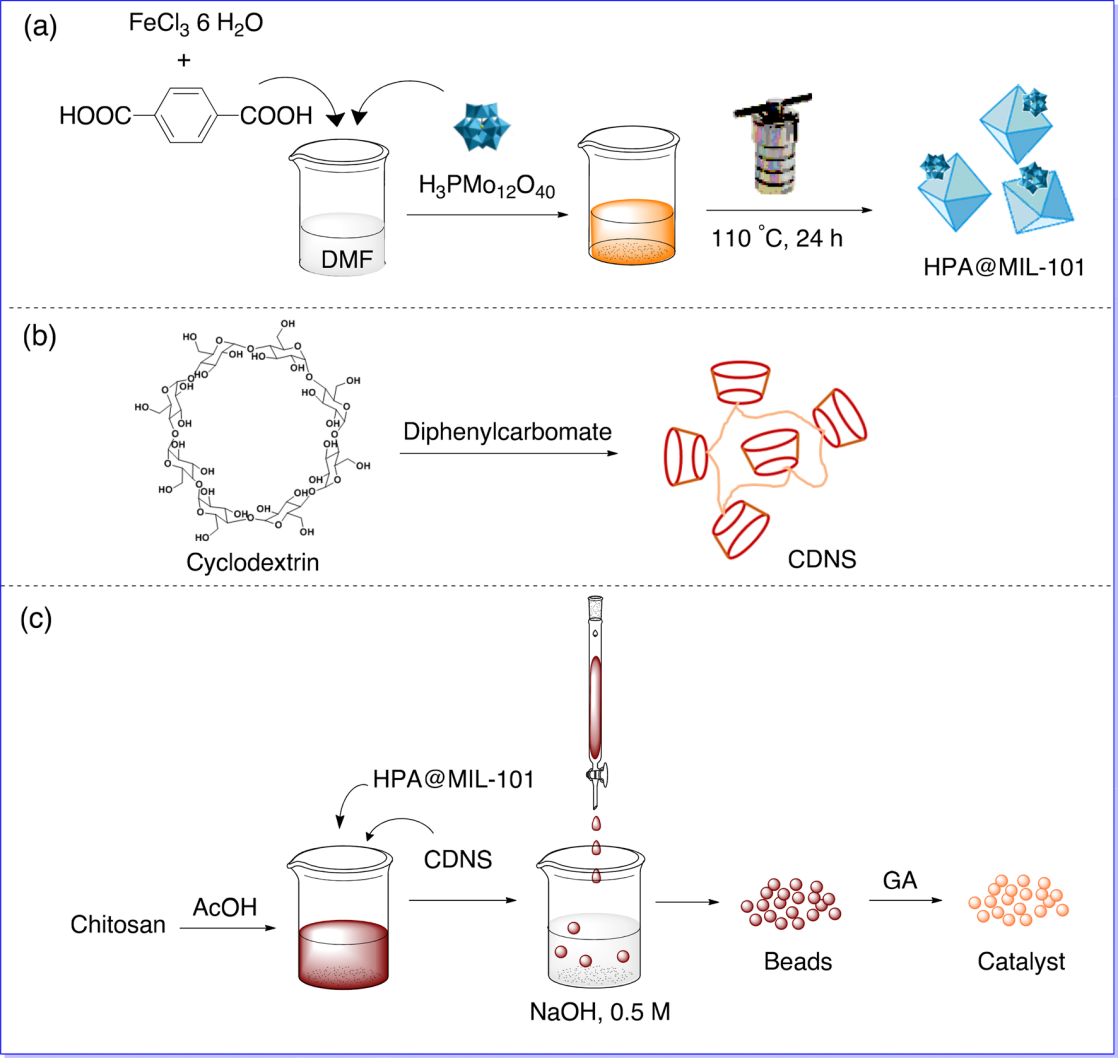
Pictorial procedure for the preparation of (**a**) HPA@MIL-101, (**b**) CDNS, and (**c**) CS-CDNS-HPA@MIL-101.

In this work, for the first time, a novel multi-task catalytic composite (CS-CDNS-HPA@MIL-101), Fig. 1, is designed and prepared that benefits from the chemistry of MOF, HPA, CDNS, and CS beads. The merits of this catalytic composite are its multi-functionality, use of bio-based components, and a small amount of HPA and MOF. The central hypothesis of our study is that the catalyst possessing both acidic and redox potential could efficiently promote alcohol oxidation and cascade alcohol oxidation–Knoevenagel condensation reactions, Scheme 1, in aqueous media under mild conditions. More importantly, the catalyst exhibited high recyclability and low HPA leaching, which makes this catalyst environmentally benign

.

## Results and discussion

### Characterization of CS-CDNS-HPA@MIL-101

**Figure 2:**
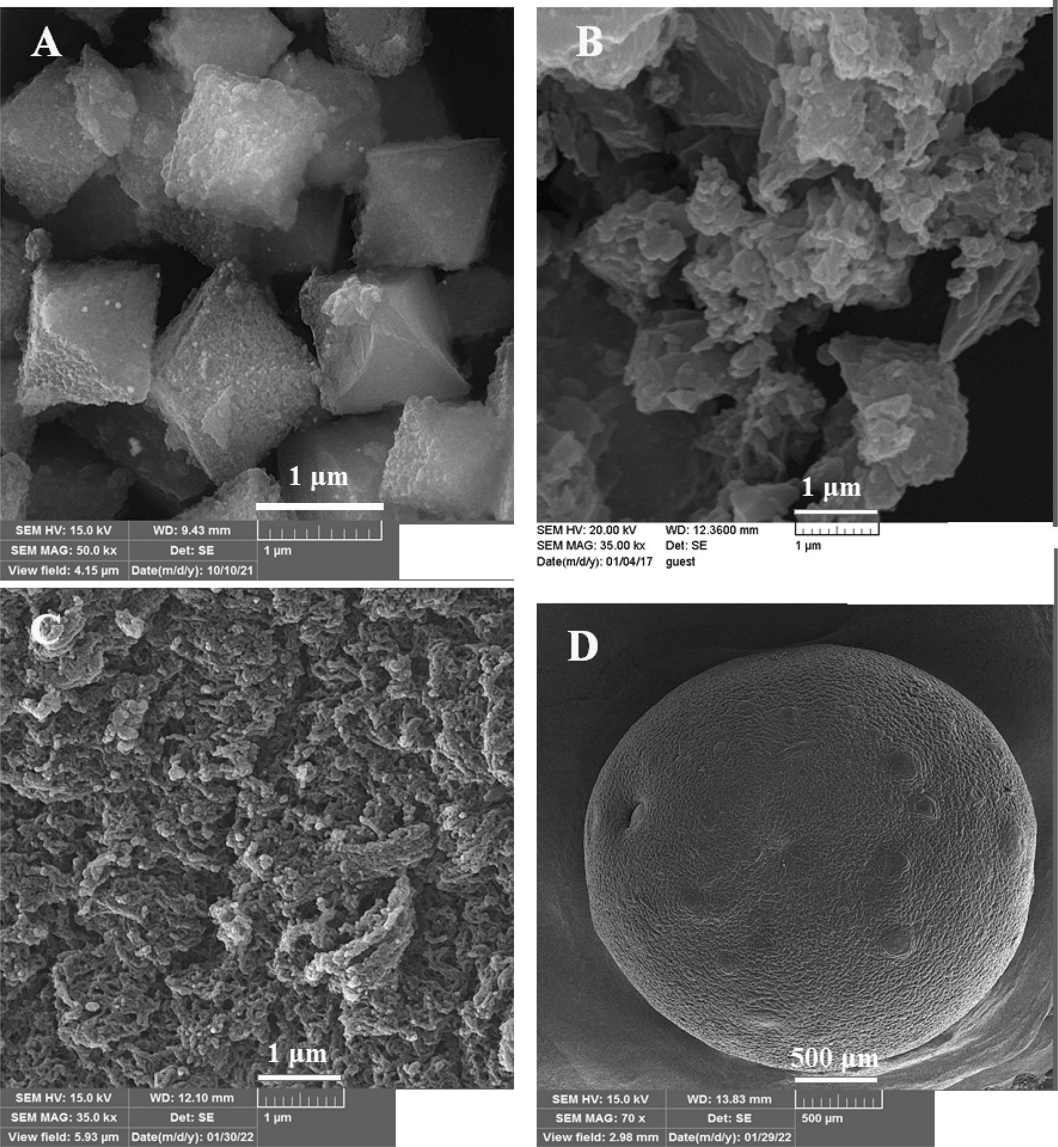
FE**-**SEM images of (**a**) HPA@MIL-101, (**b**) CDNS, (**c**) and (**d**) CS-CDNS-HPA@MIL-101**.**

The morphology of HPA@MIL-101, CDNS, and CS-CDNS-HPA@MIL-101 were evaluated with FE-SEM. Figure 2a depicts an uniform polyhedron morphology for HPA@MIL-101 with average diameter of 2 ± 0.4 μm that is similar to the reported value of MIL-101^53^. This data indicates that incorporation of low content of HPA during MIL-101 synthesis did not change the morphology of the final product. As shown in Fig. 2b, the as-prepared CDNS exhibited amorphous morphology. This observation is in good agreement with previous report^54^. FE-SEM images of CS-CDNS-HPA@MIL-101 (Fig. 2c, d), showed formation of beads with average diameter of 2 ± 0.5 mm. Additionally, FE-SEM images with high magnitude confirmed that the surface of the formed beads was rough and porous.

**Figure 3:**
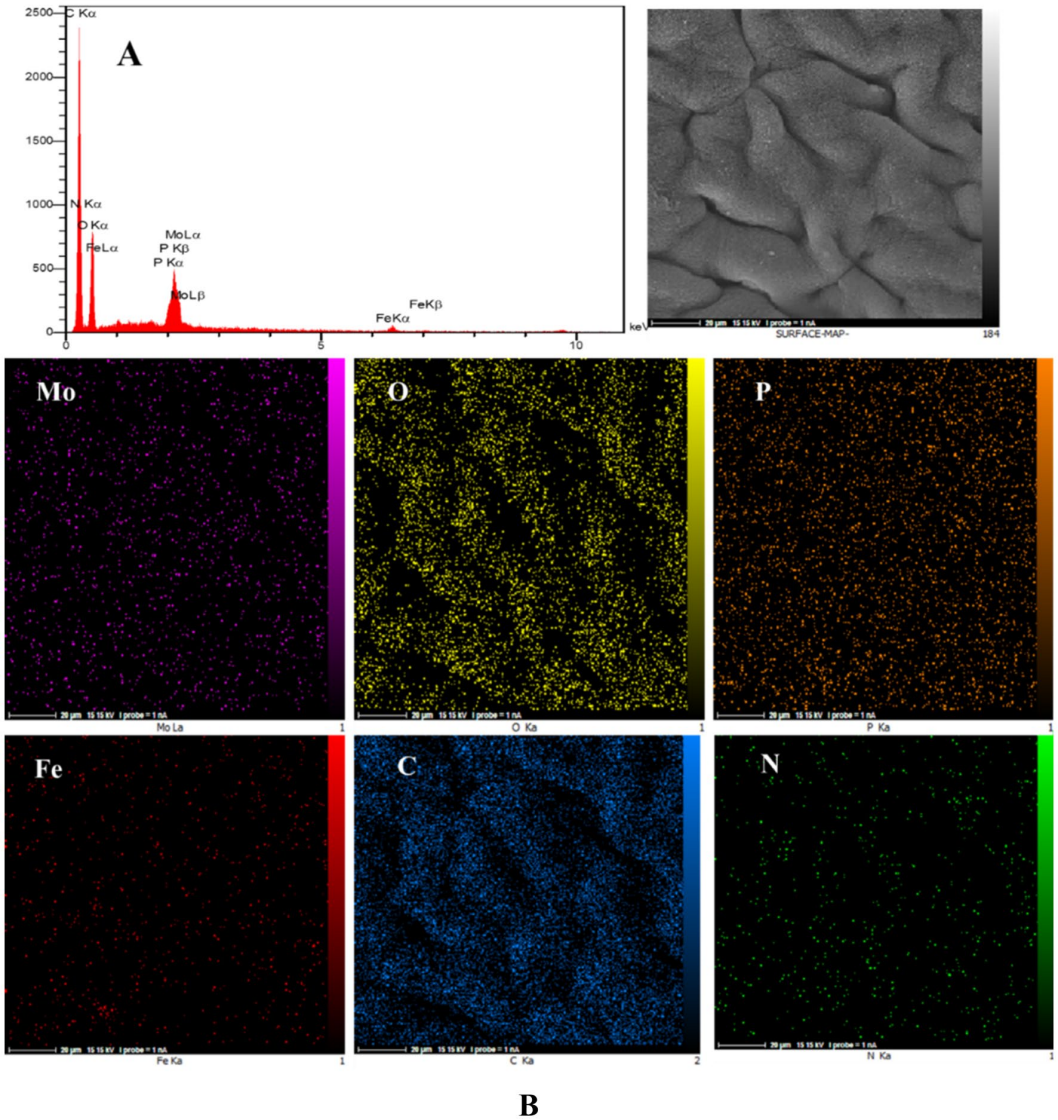
(**A**) EDS and (**B**) elemental mapping analysis of the catalyst (CS-CDNS-HPA@MIL-101).

The energy dispersive X-ray spectroscopy (EDS) and elemental mapping analyses were also carried out and demonstrated the presences of N, O, Fe, Mo, P, and C atoms in the CS-CDNS-HPA@MIL-101 (Fig. 3a). Among the detected atoms, P, O, and Mo atoms are indicative of HPA, while C, O, and N atoms are representative of CS. Furthermore, C and O atoms can be assigned to CDNS structure. Observation of Fe atom as well as O and C, is a proof for the presence of MIL-101 in the structure of the catalyst. Elemental mapping analysis, Fig. 3b, confirmed homogeneous dispersion of all atoms in CS-CDNS-HPA@MIL-101, indicating that both CDNS and HPA@MIL-101 are well-dispersed in the structure of the formed beads. The as-prepared CS-CDNS-HPA@MIL-101 consist of CS, CDNS and HPA@MIL-101.

**Figure 4:**
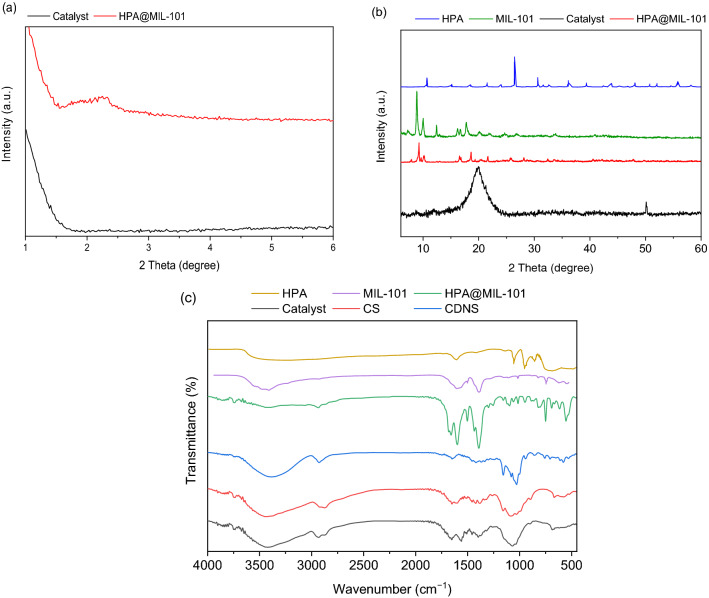
(**a**) Low-angle XRD patterns of HPA@MIL-101 and the catalyst (CS-CDNS-HPA@MIL-101), (**b**) wide-angle XRD patterns of HPA, MIL-101, HPA@MIL-101 and the catalyst, and (**c**) FTIR spectra of CS, HPA, MIL-101, CDNS, HPA@MIL-101, and the catalyst (CS-CDNS-HPA@MIL-101).

It is well-reported that CS and CDNS prepared through melt-method are amorphous and their XRD patterns consist of a broad peak at 2θ = 15–25° ^52,55^. As shown, the characteristic peaks of HPA are the peaks at 2θ = 10°, 26.4°, and 30.6°. The wide-angle XRD pattern of MIL-101 exhibited the characteristic peaks at 2θ = 9.3°, 9.9°, 16.3°, 18.7°, 21.9°, 26.8° and 28.1°. According to the literature, MIL-101 also shows a peak at 2θ =  ~ 2° in low-angle XRD^56^. Wide-angle XRD pattern of HPA@MIL-101, Fig. 4B, is identical to that of MIL-101^53^ and showed the characteristic peaks at 2θ = 9.3°, 9.9°, 13.0°,16.6°, 18.6°, 21.6°, 25.8° and 28.1° ^57^. In the low-angle XRD pattern of this sample, the characteristic peak at 2θ =  ~ 2° is assigned to the MIL-101. In fact, as a result of low content of HPA as well as its high dispersion, HPA characteristic peaks were not observed in the XRD pattern of HPA@MIL-101^58^. As shown in Fig. 4B, in the wide-angle XRD pattern of CS-CDNS-HPA@MIL-101, a broad peak in the range of 2θ = 14–25° was discerned and the characteristic peaks of HPA@MIL-101 were not detected. Moreover, the low-angle XRD pattern of the catalyst, Fig. 4A, indicated the disappearance of the characteristic peak at 2θ =  ~ 2°. According to the literature, when MOF is incorporated with another component to form a composite, the intensity of the characteristic peaks of MOF can be reduced^59^ or even the peaks can be undetectable^56,60^.

To confirm formation of HPA@MIL-101 and the catalyst, their FTIR spectrum was recorded and compared with that of CS, HPA, MIL-101 and CDNS (Fig. 4). The characteristic bands of CS appeared at 3446 cm^−1^ (–OH), 2868 cm^−1^ (–CH_2_), 1388 cm^−1^ and 1649 cm^−1^ (–C–O). FTIR spectrum of MIL-101exhibits the absorbance band at 1422, 1591 and 1651 cm^–1^ that are assigned to the asymmetric and symmetric stretching modes of the O–C=O framework^38^. The FTIR spectrum of the as-prepared CDNS (Fig. 4) is in good agreement with the literature and exhibited the absorbance bands at 3378 cm^−1^ (–OH), 2930 cm^−1^ (–CH_2_), 1649 cm^−1^ (–C–O) and 1739 cm^−1^ (–C=O)^38^. In the FTIR spectrum of HPA, the absorbance bands detected at 1062 cm^−1^ and 956 cm^−1^ are ascribed to P–O and Mo–O vibration respectively. The band observed at 847 cm^−1^ is assigned to the Mo–O–Mo vibration^61^. The absorbance bands of HPA@MIL-101 appeared at 1413, 1595 and 1660 cm^−1^ that are assigned to the asymmetrical and symmetrical stretching modes of O–C=O and 1388 cm^−1^ that is assigned to the aromatic carbon C–C vibrational mode^53^. It is worth mentioning that the characteristic bands observed at 1058 *ν*(P-Oa), 948 ν(Mo-Od), 885 ν(Mo-Ob-Mo), and 750 cm^−1^ *ν*(Mo-Oc-Mo) can be attributed to HPA, which is reported previously^62^. In the FTIR spectrum of CS-CDNS-HPA@MIL-101, all characteristic bands of the composite components were discerned, while some of them overlapped together.

**Figure 5:**
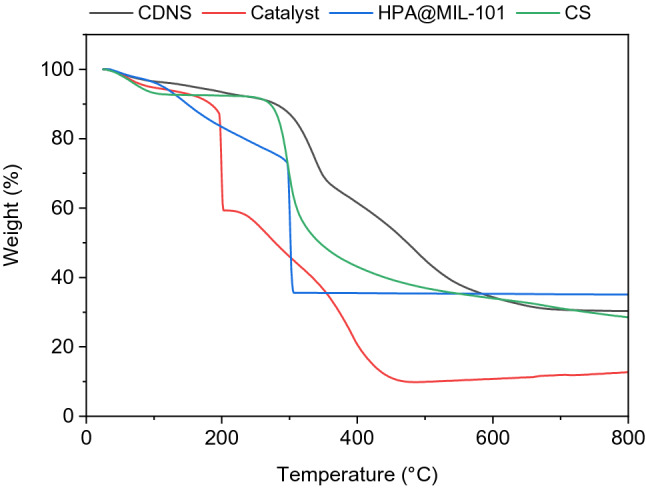
TG curves of CDNS, CS, HPA@MIL-101, and the catalyst (CS-CDNS-HPA@MIL-101).

The thermal behavior of HPA@MIL-101 and CS-CDNS-HPA@MIL-101, was evaluated using TG analysis and compared with that of CS and CDNS (Fig. 5). The thermogram of CS exhibited two weight losses at around 100 °C, dehydration, and 300 °C, CS backbone degradation. CDNS thermogram showed two weight losses due to dehydration at ~ 100 °C and CDNS degradation at 350 °C, which are in good agreement with the literature^51^. In the HPA@MIL-101 thermogram, loss of water at ~ 100 °C was observed. The weight loss detected at ~ 300 °C can be attributed to the decomposition of the organic linkers in the structure of MOF. Figure 5 shows two weight loss steps in the thermogram of CS-CDNS-HPA@MIL-101 related to the loss of water at ~ 100 °C and degradation of organic moiety (65 wt.%), i.e., CS, CDNS and the organic linkers of MOF at 200–350 °C.

NH_3_-TPD was applied to elucidate the acidity characteristic of CS-CDNS-HPA@MIL-101. The literature shows^63^ that acidic sites with different strength can be classified according to the temperature of desorbed NH_3_ peaks. In more detail, the corresponding peaks of weak, moderate and strong acidic sites appear at 100–330, 300–450 and > 450 °C respectively. In the NH_3_-TPD profiles of CS-CDNS-HPA@MIL-101, two peaks were detected, one in the range of 300–600 °C that is representative of moderate to strong acidic sites and another one in the range of 650–750 °C that is indicative of strong acidic sites (Table 1). As listed in Table 1, total acidity in the catalyst was 4900 mmol/g cat.

**Table 1:** NH_3_-TPD result for the catalyst (CS-CDNS-HPA@MIL-101).

Catalyst	Peak^a^	Acidity^b^	Peak^a^	Acidity^b^	Total acidity (mmol/g cat)
CS-CDNS-HPA@MIL-101	469	2584	470	2316	4900

^a^NH_3_ peak position.

^b^Acidity amounts (NH_3_/Cat, µmol·g^–1^).

**Figure 6:**
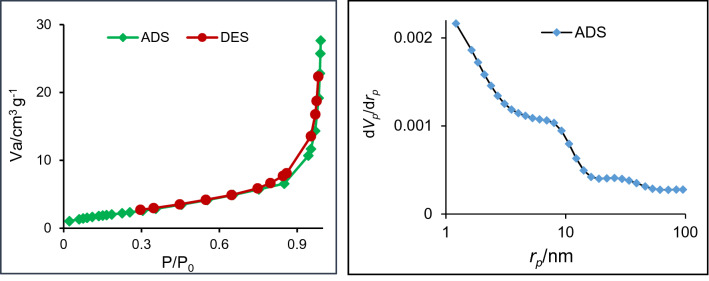
N_2_ adsorption–desorption isotherm (left) and BJH plot (right) of the catalyst.

Measurements of the textural properties of the catalyst are presented in Fig. 6. The specific surface area and pore diameter of the catalyst were ~ 9 m^2^g^−1^ and 2.4 nm, respectively. Low specific surface area is not beyond our expectations due to the presence of chitosan in the structure of the composite, which has a very low specific surface area (~ 4 m^2^ g^−1^). Specific surface area as well as other parameters, such as particle size, dispersion of the active catalytic species, the strength of the catalytic species and the number of the active sites on the catalyst can affect the catalytic activity. In fact, in determining the catalytic activity of a catalyst mostly more than one factor can be effective. The present catalyst is composed of H_3_PMo_12_O_40_@MIL-101(Fe) in the CDNS-CS bead and more precisely, the main part of the catalyst is chitosan. Notably, the specific surface area of chitosan is low, however, the catalytic activity of chitosan for many organic transformation is high^64^. This issue can be due to the various catalytic active sites on chitosan. In other word, the specific surface area is not the only determining factor on the catalytic activity. In this catalyst, the presence of MOF and HPA that is a potent catalytic species, their good dispersion and also the presence of CDNS that is a well-known phase transfer catalyst and chitosan, which is a well-established catalyst can justify high activity of the catalyst.

### Catalytic activity of CS-CDNS-HPA@MIL-101

#### Optimization of the reaction parameters

The main goal of our study was to promote alcohol oxidation and alcohol oxidation-Knoevenagel condensation reaction in aqueous media by designing a novel bi-functional catalyst, CS-CDNS-HPA@MIL-101. In this regard, we hypothesize that HPA with both acidic and redox potentials can promote alcohol oxidation reactions. Moreover, HPA and CS can catalyze Knoevenagel condensation reaction. In addition, CDNS was used in the catalyst structure, as a phase transfer agent, to facilitate reaction in aqueous media. It should be noted that the incorporation of HPA@MIL-101 into the bead could suppress HPA leaching.

First, alcohol oxidation was targeted and the reaction variables, including the amount of CS-CDNS-HPA@MIL-101 catalyst, oxidant content, reaction temperature and the nature of the solvent were optimized to achieve the highest conversion and yield. In this context, oxidation of benzyl alcohol was selected as a model oxidation reaction for performing optimization experiments.

#### Effect of catalyst amount

**Figure 7:**
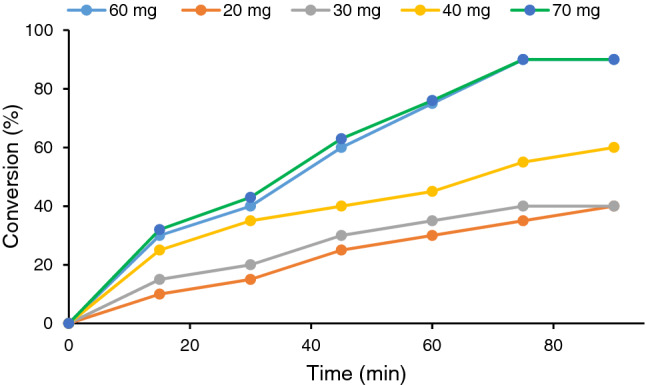
Screening the effect of the catalyst loading on the conversion of the model alcohol oxidation reaction. The reaction was performed using water as solvent and 2 mmol H_2_O_2_ at 55 °C.

To study the effect of the catalyst loading, the model reaction was repeated in the presence of various dosages of CS-CDNS-HPA@MIL-101 and the progress of each reaction was monitored precisely, Fig. 7. Comparison of the conversions of the reactions implied that increase of CS-CDNS-HPA@MIL-101 content from 20 to 30 mg, led to slight increase of the reaction conversion. However, further increase of the catalyst content to 40 mg resulted in more pronounced increment of the reaction conversion. This trend was followed upon increase of this parameter to 60 mg and the reaction conversion reached to 90% after 75 min. Further increase of CS-CDNS-HPA@MIL-101 loading to 70 mg, however, had insignificant effect on the reaction conversion. More precisely, at the start of the reaction, conversion in the presence of 70 mg catalysts was ~ 2% higher than that of 60 mg catalyst, however, after 60 min, similar conversions were observed for the reactions in the presence of 60 and 70 mg CS-CDNS-HPA@MIL-101. Considering these results, the optimum amount of CS-CDNS-HPA@MIL-101 loading was selected as 60 mg per 1 mmol of the substrate.

#### Effect of oxidant content

**Figure 8:**
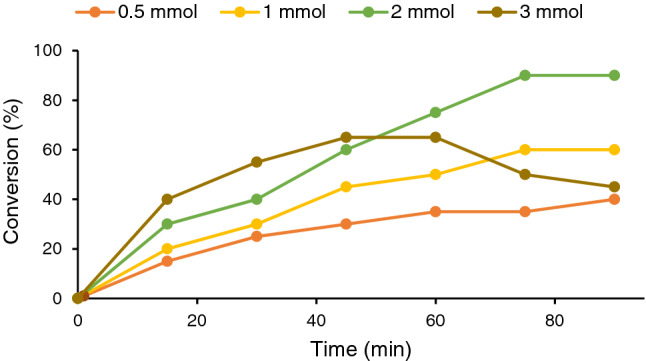
Screening the effect of oxidant amount on the conversion of the model alcohol oxidation reaction. The reaction was performed using water as solvent and 60 mg CS-CDNS-HPA@MIL-101at 55 °C.

Next, the amount of the oxidant, H_2_O_2_, was optimized by studying the effect of different amounts of the oxidant (0.5–3 mmol) on the conversion of the catalyst, Fig. 8. As displayed, use of low content of the oxidant (0.5 mmol) led to low conversion. Upon increase of the oxidant amount from 0.5 to 2 mmol, the reaction conversion improved gradually to reach 90% after 75 min. It is worth noting that further increase of this value to 3 mmol had a detrimental effect on the reaction conversion. Actually, use of high content of oxidant promoted oxidation of benzyl alcohol to benzoic acid. According to these experiments, the optimum value for the oxidant amount was 2 mmol per each mmol of the substrate.

#### Effect of reaction temperature

**Figure 9:**
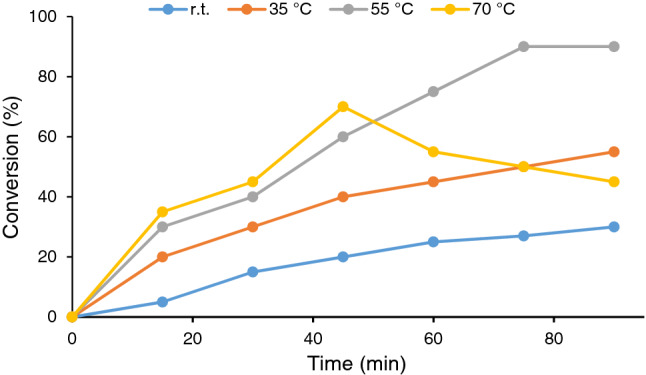
Screening the effect of temperature on the conversion of the model alcohol oxidation reaction. The reaction was performed using water as solvent, 60 mg CS-CDNS-HPA@MIL-101 and 2 mmol H_2_O_2_.

The reaction temperature is another important parameter that is optimized in this work. Figure 9 shows the conversions of the model reactions at different temperatures, ambient temperature (25 °C) to 70 °C. These data indicated that increase of the reaction temperature from ambient temperature (25 °C) to 55 °C increased the reaction conversion remarkably. However, as further increase of this parameter led to the oxidation of benzyl alcohol to benzoic acid, lower yield of benzaldehyde was observed. In fact, the results confirmed that both reaction temperature and the amount of the oxidant could affect the selectivity of alcohol oxidation and their optimization is imperative to reach high conversion towards formation of aldehyde.

#### Effect of reaction solvent

As mentioned, with the goal of designing a catalyst that could promote alcohol oxidation and alcohol oxidation-Knoevenagel condensation in aqueous media, CDNS was incorporated in the structure of the catalyst to serve as a phase transfer agent. Hence, the activity of the model reaction was first examined in water as solvent. The result showed that in the aqueous media and using 60 mg CS-CDNS-HPA@MIL-101 catalyst and H_2_O_2_ (2 mmol), at 55 °C, 90% conversion of the desired product was obtained. Notably, using EtOH that is a protic solvent, similar results were observed. The performance of the catalyst was also studied in THF and CH_3_CN and it was found that in those aprotic solvents, lower conversions and yields were achieved. The data are summarized in Table S1.

#### Generality study

The optimization experiments confirmed that CS-CDNS-HPA@MIL-101 could efficiently promote the model alcohol oxidation reaction under the optimum reaction conditions. To confirm the generality of the developed protocol, examining of other substrates with different properties was imperative. As listed in Table S2, various alcohols with different electronic and steric properties could undergo oxidation reaction under the optimum reaction conditions to furnish the corresponding aldehydes in high to excellent yields. Comparison of the yields of the substrates, indicated that oxidation of aliphatic substrates was less efficient than benzyl alcohol derivatives. Moreover, it was observed that the presence of the electron-windrowing functional groups on the aromatic ring was beneficiary and higher yields were achieved in the cases of substrates with less electronic density.

#### Alcohol oxidation-Knoevenagel condensation

Confirming high catalytic activity of CS-CDNS-HPA@MIL-101 for alcohol oxidation reaction, its performance for the cascade oxidation–Knoevenagel condensation reaction was also studied. In fact, it was assumed that the redox potential of the catalyst could promote alcohol oxidation reaction, while the presence of CS and CDNS as well as the acidic characteristic of HPA@MIL-101 could catalyze Knoevenagel condensation reaction. To validate this assumption, cascade oxidation-Knoevenagel condensation reaction of benzyl alcohol and malononitrile was first conducted under the aforesaid optimum conditions. Gratifyingly, it was found that under the so-called conditions, the desired product was achieved in 90% yield. Motivated by this result, cascade oxidation-Knoevenagel condensation reaction of various alcohols was carried out to assay the generality of this protocol, Table S3. As tabulated, various alcohols with different steric and electronic characteristic tolerated this cascade reaction to give the corresponding product in high to excellent yields. Similar to the alcohol oxidation reaction, the presence of aromatic ring on the backbone of the substrate led to the higher yields of the products.

#### Control catalysts

HPA in the structure of CS-CDNS-HPA@MIL-101 possesses redox potential and was utilized for catalyzing alcohol oxidation. To render HPA heterogeneous, it was encapsulated in MIL-101 and the resultant HPA@MIL-101 was then included in the CS-CDNS bead structure. In fact, it was postulated that interactions between HPA@MIL-101 and the functional groups on CS and CDNS can contribute to embed HPA@MIL-101 in the bead structure. In catalyst design, two roles were perceived for CDNS, as a phase transfer agent and as a bio-based catalyst for promoting Knoevenagel condensation. In more detail, encapsulation of hydrophobic substrates in the CDNS pores can facilitate their transferring to aqueous media and consequently enhance the reaction yield. On the other hand, the multiple –OH functionalities on CDNS can activate the substrate and promote Knoevenagel condensation. Another reason for incorporation of HPA@MIL-101 in CS-CDNS bead was the catalytic nature of CS and its potential for promoting Knoevenagel condensation. Furthermore, the cross-linked CS-CDNS beads were robust and could be easily separated from the reaction media. Notably, it was assumed that possible synergistic effects among the composite components can improve the catalytic activity of the composite compared to individual components. To validate the aforementioned assumptions, several control catalysts (HPA@MIL-101, CS-HPA@MIL-101, CDNS-HPA, CS-HPA, CS-CDNS-HPA) have been prepared and their activity for the model alcohol oxidation-Knoevenagel condensation reaction was examined under the optimum reaction conditions. The results, Table 2, designated that under the aforementioned conditions, CS-HPA and CDNS-HPA could catalyse the reaction to furnish the desired alcohol oxidation-Knoevenagel condensation product in 55% and 45% yield respectively. It is supposed that apart from HPA, the functionalities on CS and CDNS can participate in the catalysis. Next, to investigate the role of MIL-101 in the catalysis, a MIL-101-free control catalyst, CS-CDNS-HPA, was prepared and examined for the model alcohol oxidation-Knoevenagel condensation reaction. As shown in Table 2, the catalytic activity of this sample was higher than the aforesaid control catalyst and lower than that of CS-CDNS-HPA@MIL-101, confirming the role of MIL-101 in the catalysis. To further affirm contribution of MIL-101 to the catalysis, HPA@MIL-101 control catalyst was prepared and its activity for the model reaction was appraised. As listed, using this control catalyst, moderate reaction yield (65%) was achieved that was higher compared to CS-HPA, CDNS-HPA and CS-CDNS-HPA. This observation showed that MIL-101 not only is a potent support, but also can contribute to the catalysis. To demonstrate the role of CDNS in the catalysis, a CDNS-free control catalyst, CS-HPA@MIL-101, was examined for the model reaction and the result showed that this catalyst led to the desired product in 70% yield. As the activity of this control catalyst was lower than that of CS-CDNS-HPA@MIL-101, it can be deduced that CDNS play an important role in the catalysis. As mentioned before, the main role of CDNS is shuttling the substrates in the aqueous media. Noteworthy, the catalytic activity of all of the aforementioned control catalysts was inferior to that of CS-CDNS-HPA@MIL-101, confirming that hybridization of CS, CDNS and HPA@MIL-101 is beneficiary for the catalysis.

**Table 2:** The activity of the control catalysts and CS-CDNS-HPA@MIL-101 for the model alcohol oxidation-Knoevenagel condensation reaction.

Entry	Catalyst	Yield (%)
1	CS-CDNS-HPA@MIL-101	90
2	CS-HPA	55
3	CDNS-HPA	45
4	CS-CDNS-HPA	61
5	HPA@MIL-101	65
6	CS-HPA@MIL-101	70

Reaction conditions: alcohol (1 mmol), H_2_O_2_ (30%, 0.6 mmol) and CS-CDNS-HPA@MIL-101 (60 g) were mixed in H_2_O (7 mL) and then, malononitrile (1.2 mmol) was added and the reaction was proceeded at 55 °C for 1.5 h.

### Recyclability of CS-CDNS-HPA@MIL-101

**Figure 10:**
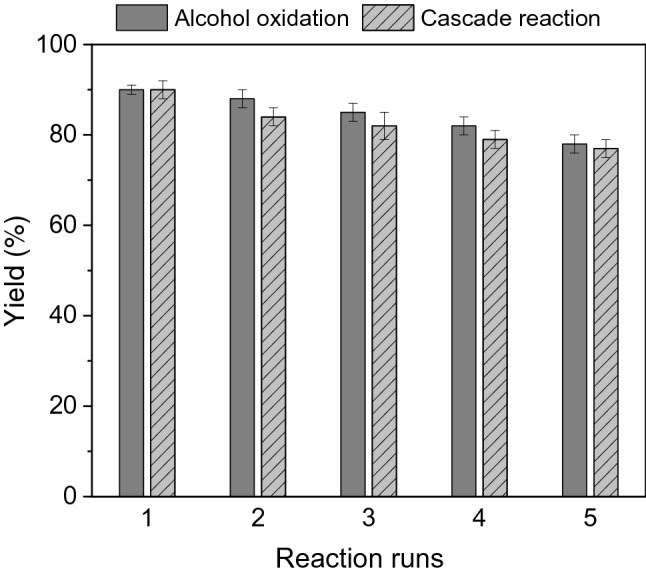
Recyclability of CS-CDNS-HPA@MIL-101 for the model alcohol oxidation and cascade alcohol oxidation-Knoevenagel condensation reaction. Reaction conditions for alcohol oxidation reaction: mixing of alcohol (1 mmol), CS-CDNS-HPA@MIL-101 (60 mg) and H_2_O_2_ (2 mmol) at 55 °C. Reaction conditions for cascade alcohol oxidation-Knoevenagel condensation reaction: alcohol (1 mmol), H_2_O_2_ (30%, 0.6 mmol) and CS-CDNS-HPA@MIL-101 (60 g) were mixed in H_2_O (7 mL) and then, malononitrile (1.2 mmol) was added and the reaction was stirred at stirred at 55 °C for 1.5 h.

Recyclability of a heterogeneous catalyst is an important feature, determining its potential for commercial and large-scale uses. Considering high solubility of HPA in conventional solvents, some HPA-based heterogeneous catalyst showed poor recyclability. To appraise the recyclability of CS-CDNS-HPA@MIL-101, its performance for five consecutive runs for both model alcohol oxidation and cascade alcohol oxidation-Knoevenagel condensation reactions under the optimum conditions was evaluated. To this purpose, after the first run of the reaction the catalyst was separated, washed with EtOH and dried at 70 °C overnight. Then the recovered catalyst was reused for the next reaction run. As shown in Fig. 10, for both reactions, slight loss of the catalytic activity was observed upon each run of recycling and the reaction conversion decreased from 90 to 78% for benzyl alcohol oxidation reaction and 90–77% for cascade benzyl alcohol oxidation-Knoevenagel condensation reaction on the fifth run of the reactions.

To assay the origin of loss of the activity of CS-CDNS-HPA@MIL-101 upon recovery-reuse cycle, the recovered catalyst after the fifth run of alcohol oxidation-Knoevenagel condensation was analysed via FTIR spectroscopy. In the FTIR spectrum of the recycled CS-CDNS-HPA@MIL-101, Figure S1, the distinguished absorbance bands of fresh CS-CDNS-HPA@MIL-101 are detectable, confirming that CS-CDNS-HPA@MIL-101 was stable upon several runs of recycling. It is worth mentioning that in the FTIR spectrum of the recycled catalyst, an additional band at 2212 cm^−1^ can be discerned that is ascribed to the –CN functionality, indicating deposition of malononitrile and/or cascade reaction product on the catalyst. As the deposited chemicals can hinder the accessibility of the substrates to the active sites of CS-CDNS-HPA@MIL-101, it can play a role in deactivation of the catalyst. Another important factor that can affect the catalytic activity is leaching of HPA. To elucidate whether several runs of recycling can trigger HPA leaching, ICP analysis of the recycled CS-CDNS-HPA@MIL-101 was conducted. Gratifyingly, the leaching of HPA after five runs of recycling was insignificant (only 1 wt.% of the initial loading), implying that incorporation of HAP in the composite could efficiently suppress its leaching.

**Figure 11:**
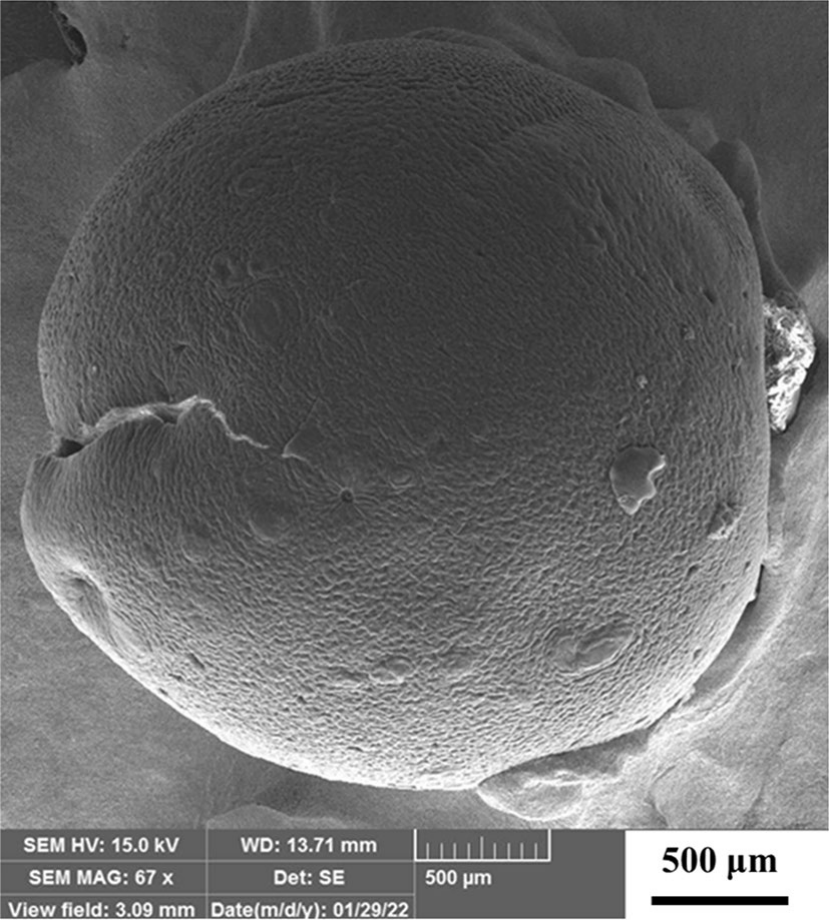
FE**-**SEM image of the recycled catalyst after five runs of cascade alcohol oxidation-Knoevenagel condensation reaction. Reaction conditions alcohol (1 mmol), H_2_O_2_ (30%, 0.6 mmol) and CS-CDNS-HPA@MIL-101 (60 g) were mixed in H_2_O (7 mL) and then, malononitrile (1.2 mmol) was added and the reaction was stirred at stirred at 55 °C for 1.5 h.

Morphological study of the recycled catalyst after five runs of the cascade alcohol oxidation-Knoevenagel condensation reaction under the optimum reaction condition, Fig. 11, indicated that even after several recovery and reusing runs, the morphology of the catalyst is similar to the fresh catalyst and the catalyst preserve its spherical morphology. However, in some cases the beads showed some crakes.

### Hot filtration test

In the heterogeneous catalysis, two possible routes can be followed. In the first route, which is referred as true heterogeneous catalysis, the catalytic species are immobilized on the support in the course of the reaction. While in the second route, the stabilized catalytic species leaches in the reaction media during the reaction and then re-deposits on the support. Hence, to elucidate whether the catalysis is truly heterogeneous, Hot-filtration test is used^65^, in which the reaction is halted after a short reaction time, the catalyst is removed from the reaction media and the progress of the reaction is monitored over the time. In the case of true heterogeneous catalysis, it is expected that the reaction does not proceed after separation of the catalyst. In this study, to elucidate the nature of CS-CDNS-HPA@MIL-101 catalysis, hot filtration test was performed for both CS-CDNS-HPA@MIL-101-catalysed benzyl alcohol oxidation and the cascade alcohol oxidation-Knoevenagel condensation under the optimum reaction conditions. Monitoring of the conversion of both reactions (Figure S2) indicated that in the presence of the catalyst, the reactions proceeded to furnish 52% aldehyde after 30 min and 49% benzylidene malononitrile after 15 min. Upon removal of CS-CDNS-HPA@MIL-101 from the reaction media, no noticeable change in the conversion of both reactions was observed over the time. These findings imply the true heterogeneous nature of catalysis.

### Plausible mechanism for alcohol oxidation-Knoevenagel condensation

**Figure 12:**
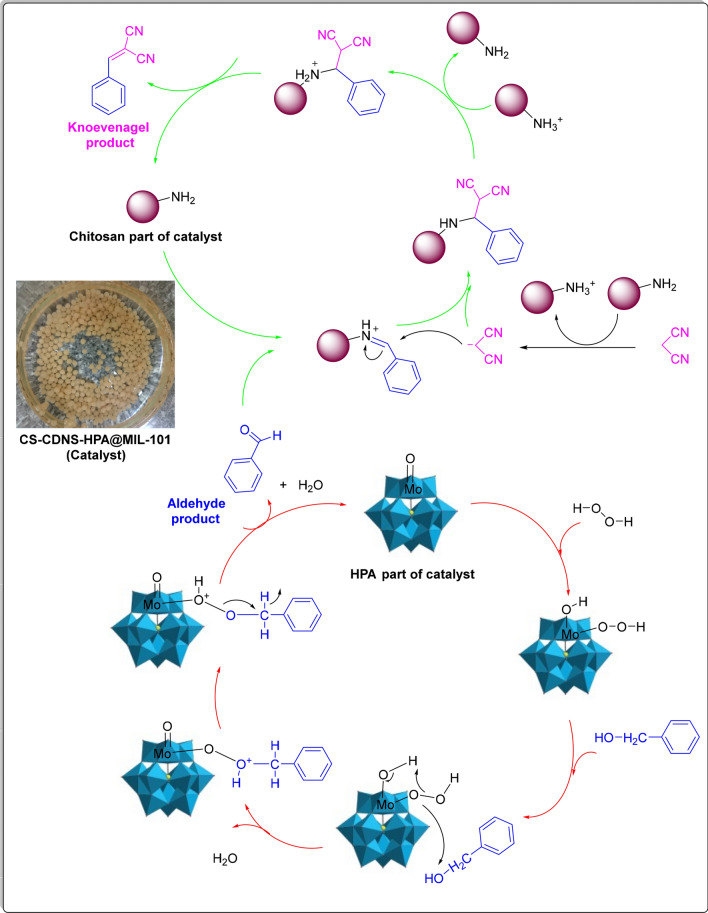
The plausible mechanism for the cascade alcohol oxidation-Knoevenagel condensation reaction highlighting the catalyst effect.

The selected cascade reaction in our study involves two consecutive steps, alcohol oxidation and Knoevenagel condensation, which is illustrated in Fig. 12. According to the literature^66^, in the first step, one proton of H_2_O_2_ is transferred to one of the oxygen atoms in the MoO_2_ unit in HPA and the formed HO_2_^−^ is coordinated to the Lewis-acidic metal center to generate peroxo intermediate, which oxidizes alcohol to furnish the corresponding aldehyde and water. In the next step, the generated aldehyde takes part into the Knoevenagel condensation. In this regard, the catalyst activates malononitrile to form an activated methylene compound. Simultaneously, the catalytically active sites in the bead can activate the aldehyde to form an active intermediate, which then tolerates reaction with the as-prepared activated methylene compound to give the Knoevenagel condensation product as well as the catalyst (Fig. 12)^67^.

### Comparative study

Alcohol oxidation-Knoevenagel condensation reaction is an important cascade reaction that can be applied for the synthesis of more complicated organic compounds. To date various catalysts have been developed for promoting this reaction under different reaction conditions. Table 3 summarized the performance of the model reactions with some randomly selected catalysts to assay the catalytic activity of CS-CDNS-HPA@MIL-101 in comparison with the previously reported ones. Comparison of the yield of the model product as well as the reaction conditions affirms that some catalysts are inefficient and led to scant yield of the desired product (Table 3, entries 2, 4, and 7). Although some other catalysts, such as NH_2_-MIL-101(Fe) and Zr-MOF-NH_2_ resulted in moderate to high yields of the desired product, use of organic and toxic solvents as well as long reaction time rendered them less attractive. Taking the data in Table 3 into account, the catalytic activity of CS-CDNS-HPA@MIL-101 is superior to or equivalent to the reported catalysts so far. These data confirm that the designed catalyst can be considered as an efficient catalyst for promoting alcohol oxidation-Knoevenagel condensation reactions. It is worth noting that there are defiantly some protocols that may led to the higher yields of the desired products in even milder conditions. In fact, our random comparison is just for indicating that the activity of CS-CDNS-HPA@MIL-101 is comparable with some reported catalysts.

**Table 3:** The activity of CS-CDNS-HPA@MIL-101, the designed catalyst in this work, for alcohol oxidation-Knoevenagel condensation reaction in comparison with the reported catalysts in literature.

Entry	Catalyst	Catalyst amount	Reaction conditions	Time (h)	Benzylidene malononitrile (yield %)	References
1	Cu_3_TATAT-3^a^	8 mol%	CH_3_CN/O_2_/75 °C	12	95	^68^
2	NH_2_-MIL-125(Ti)	20 mg	O_2_/light irradiation	40	3.3	^69^
3	NH_2_-MIL-101(Fe)	20 mg	O_2_/light irradiation	40	72	^69^
4	Ti-MOF-NH_2_	100 mg	*p*-Xylene/O_2_/UV irradiation	48	32	^70^
5	Zr-MOF-NH_2_	100 mg	*p*-Xylene/O_2_/UV irradiation	48	91	^70^
6	UoB-2^b^	2 mol%	Solvent-free EtOH/TBHP	1.5	94	^71^
7	NH_2_-UiO-66(Zr)	20 mg	O_2_/light irradiation	40	4.6	^69^
8	Fe_3_O_4_@SiO_2_@PEI@Ru(OH)_X_	100 mg	O_2_/110 °C for alcohol oxidation step	22	90.2	^72^
9	CS-CDNS-HPA@MIL-101	60 mg	H_2_O/H_2_O_2_/55 °C	1.5	90	This work

^a^bifunctional copper metal organic framework

^b^Ni-based metal–organic framework.

## Experimental section

The chemicals used for this research as well as the detail of the instruments are presented in supporting information.

### Preparation of HPA@MIL-101

To prepare HPA@MIL-101, FeCl_3_·6H_2_O (7 mmol) and H_2_BDC (7 mmol) were dissolved in DMF (70 mL) and mixed at ambient temperature (25 °C) for 30 min. HPA (0.12 mmol) was slowly added to the prepared solution and the mixture was stirred at ambient temperature for 2 h and then transferred into a Teflon-lined hydrothermal reactor and heated at 110 °C for 24 h. At the end, the reactor was cooled and the reaction product was isolated from solution via centrifugation. The obtained precipitate washed repeatedly with MeOH and dried in vacuum at 90 °C. ICP analysis showed that the content of HPA in HPA@MIL-101 was 6.6 wt.%.

### Preparation of CDNS

CDNS was prepared through our previously reported method^51^. Briefly, diphenyl carbonate (8 mmol) was added to a beaker and heated on a hot plate. Then, β-CD (1 mmol) was slowly added to the melted diphenyl carbonate and the mixture was stirred vigorously at 120 °C overnight to obtain a white solid. Subsequently, the beaker was cooled and the obtained CDNS was crushed to powder. Then, the prepared CDNS was purified by washing repeatedly with acetone and distilled water, followed by Soxhlet with ethanol around 12 h. The pure CDNS was then dried at ambient temperature (25 °C).

### Preparation of CS-CDNS-HPA@MIL-101

To prepare the composite bead, to a solution of CS (1 g) in acetic acid (50 mL, 2 w/v%), the as-prepared CDNS (0.33 g) and HPA@MIL-101 (0.33 g) were added. The mixture was stirred for 3 h to achieve a homogeneous suspension. Then, the obtained suspension was transferred into a burette and gently dropped into a solution of NaOH (0.5 mol L^–1^) to form fine beads and they were kept in the basic solution overnight. To covalently crosslink the beads, they were collected, rinsed with distilled water, and then introduced into a solution of GA (5 w/v% in EtOH) and stirred at 70 °C for 13 h. Finally, the covalently cross-linked beads were collected, rinsed with EtOH, and dried at ambient temperature, 25 °C (Fig. 1).

### Alcohol oxidation

To examine the activity of CS-CDNS-HPA@MIL-101 for alcohol oxidation, alcohol (1 mmol), CS-CDNS-HPA@MIL-101 (60 mg) and H_2_O_2_ (2 mmol) were mixed in H_2_O at 55 °C and the reaction progress was monitored by Thin-layer chromatography (TLC). Upon completion of the oxidation reaction, CS-CDNS-HPA@MIL-101 was removed from the reaction medium by centrifugation and the solvent of the filtrate was evaporated to achieve the product. The yield of the reaction was measured by gas chromatography (GC) and the separated catalyst was washed with EtOH, dried at 70 °C overnight and reused for the next reaction run.

### Cascade alcohol oxidation–Knoevenagel condensation reaction

The activity of CS-CDNS-HPA@MIL-101 was evaluated for cascade alcohol oxidation–Knoevenagel condensation (Scheme 1). To perform the reaction, a solution of alcohol (1 mmol), H_2_O_2_ (30%, 0.6 mmol) in H_2_O (7 mL) and CS-CDNS-HPA@MIL-101 (60 g) were placed in a round-bottomed flask and then stirred at 55 °C. Subsequently, malononitrile (1.2 mmol) was added, the reaction mixture was stirred and the progress of the reaction was monitored by TLC. Upon completion of the reaction, CS-CDNS-HPA@MIL-101 was separated and recovered by washing with EtOH and drying at 70 °C overnight. The recovered CS-CDNS-HPA@MIL-101 was used for the next run of the cascade reaction. Knoevenagel product was obtained after evaporation of the solvent and silica gel column chromatography with petroleum ether-EtOAc (5: 1), Figures S3–S12.

## Conclusions

In designing the new generation of catalyst, the multi-component catalyst benefiting from multi-functional activity addresses the requirements of green chemistry principles in a wide range of organic reactions is introduced and used for catalyzing both alcohol oxidation and cascade alcohol oxidation-Knoevenagel condensation reaction in aqueous media. We propose an easy and efficient synthesis process for the preparation of the catalyst (CS-CDNS-HPA@MIL-101). The physico-chemical characteristics of the catalyst were studied using various techniques and show a bead where the components are well-dispersed. Our finding highlights the high catalytic activity toward alcohol oxidation and cascade alcohol oxidation-Knoevenagel condensation reactions. This behavior indicates the CDNS acted mainly as a phase transfer agent, HPA served as a bifunctional catalyst with both acidic and redox potential, and amino groups on CS acted as basic catalysts. It should be noted that the encapsulation of HPA in MIL-101 and also the incorporation of HPA@MIL-101 in the structure of the bead could suppress HPA leaching and result in good recyclability of the catalyst. More importantly, the catalysis was truly heterogeneous and did not follow the leaching-deposition cycle in the course of catalysis.

## Supplementary Information

Supplementary Information.

## Data Availability

The datasets used and/or analyzed during the current study are available from the corresponding author on reasonable request.
