# Supplementary material for: Chitosan bead containing metal–organic framework encapsulated heteropolyacid as an efficient catalyst for cascade condensation reaction
Source: Sci Rep. 2023 Feb 16;13:2797. doi: 10.1038/s41598-023-29548-2 (PMC9935902; doi:10.1038/s41598-023-29548-2)
Supplement: Supplementary file 1 — Supplementary Information. [file 41598_2023_29548_MOESM1_ESM.docx]

**Supplementary information**

**Chitosan bead containing metal-organic framework encapsulated heteropolyacid as an efficient catalyst for cascade condensation reaction**

Samahe Sadjadi^1^*, Neda Abedian-Dehaghani^2^, Abolfazl Heydari^3^, Majid M. Heravi*^2^

## Chemicals

Iron(III) chloride hexahydrate (FeCl_3_‧6H_2_O), terephthalic acid (H_2_BDC), phosphomolybdic acid (HPA, H_3_PMo_12_O_40_), β-cyclodextrin (β-CD), diphenyl carbonate, glutaraldehyde (GA), malononitrile, hydrogen peroxide (H_2_O_2_, 30%), alcohols, acetic acid, sodium hydroxide (NaOH), dimethylformamide (DMF), ethyl acetate (EtOAc), and methanol (MeOH) were purchased from Sigma-Aldrich and used as received.

## Apparatus

Vista-pro instrument was applied for performing Inductively Coupled Plasma (ICP) analysis and measuring the HPA loading. To assess the thermal stability of CS-CDNS-HPA@MIL-101, thermogravimetric analysis (TGA) was conducted using METTLER TOLEDO device under nitrogen atmosphere and heating rate of 10 °C min^-1^. To study the crystalline phase of the catalyst, X-ray diffraction (XRD) pattern of CS-CDNS-HPA@MIL-101 was recorded via Siemens, D5000 equipped with Cu Kα radiation. The chemical structure of the catalyst was studied by Fourier transform infrared (FTIR) spectrum recorded on PERKIN-ELMER-Spectrum 65 apparatus (scan time of 1 s and spectral resolution of 2 cm^-1^, using KBr pellet). Morphology of CS-CDNS-HPA@MIL-101 was investigated by Field emission scanning electron microscopy (FE-SEM, MIRA3TESCAN-XMU)MIRA3TESCAN-XMU instrument). Energy dispersive spectroscopy (EDS) and elemental mapping analysis were performed via MIRA3TESCAN-XMU)MIRA3TESCAN-XMU device. The specific surface area (S_BET_) and total pore volume (V_P_) of the as-prepared catalyst, was estimated using Brunauer-Emmett-Teller (BET) method using BELSORP MINI II, BEL device with pre-heating at 150 °C for 3 h. Temperature programmed desorption of ammonia (NH_3_-TPD was utilized for measuring the acidity of the composite. To conduct this analysis, Chemisorption Analyzer, NanoSORD (made by Sensiran Co., Iran) with heating ramp rate of 20 °C/min in temperature range of 25-800 °C was applied.

## Control catalysts synthesis

Additionally, CS-CDNS-HPA, HPA@MIL-101, CS-HPA@MIL-101, CDNS-HPA, and CS-HPA were prepared as control catalysts and their activity was compared with that of CS-CDNS-HPA@MIL-101. To prepare CS-HPA@MIL-101, the same procedure used for the synthesis of CS-CDNS-HPA@MIL-101 was employed, except CDNS was not added. CDNS-HPA and CS-HPA were prepared through simple wet-impregnation method. Typically, HPA was dissolved in distilled water and added dropewisely to the suspension of CDNS or CS in water. The mixture was stirred at ambient temperature overnight and then, the solid was separated and washed with distilled water and dried at 40 °C overnight. CS-CDNS-HPA was also prepared through the same procedure used for the synthesis of the catalyst, expect, HPA was not encapsulated in MIL-101.

**Figure S1.** FTIR spectra of fresh and recycled CS-CDNS-HPA@MIL-101 after five runs of the model alcohol oxidation-Knoevenagel condensation. Reaction conditions: alcohol (1 mmol), H_2_O_2_ (30%, 0.6 mmol) and CS-CDNS-HPA@MIL-101 (60 g) were mixed in H_2_O (7 mL) and then, malononitrile (1.2 mmol) was added and the reaction was stirred at stirred at 55 °C for 1.5 h.


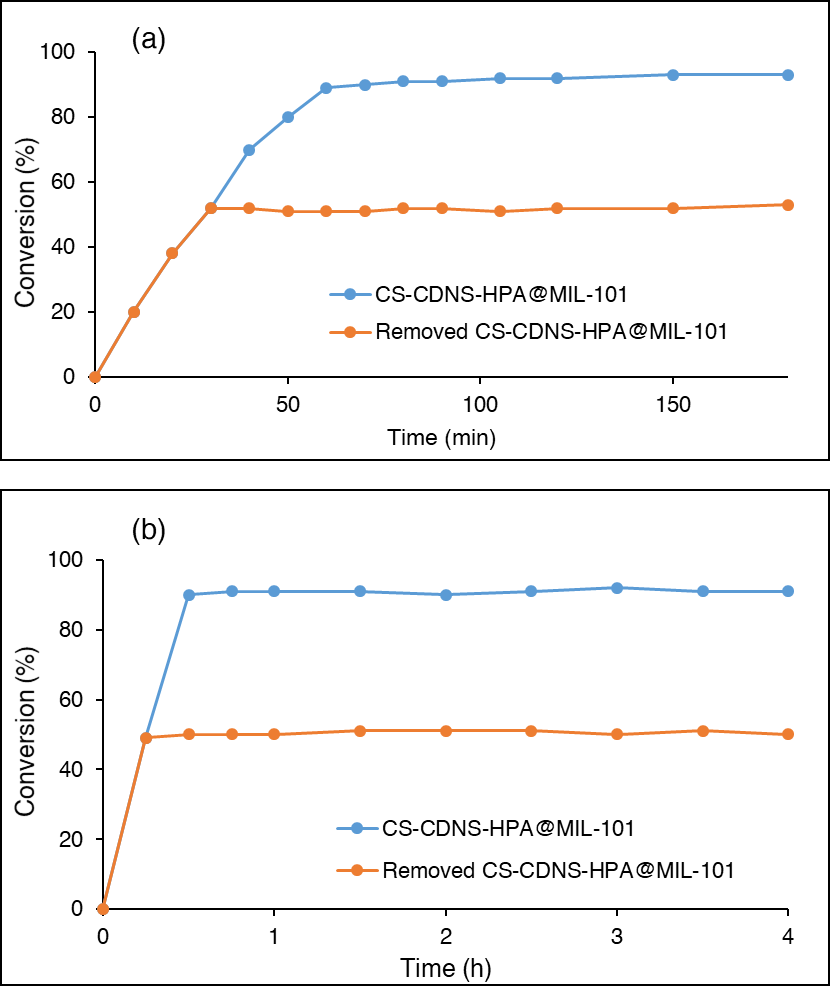


**Figure S2.** The results of hot-filtration test for (a) model alcohol oxidation and (b) the cascade alcohol oxidation-Knoevenagel condensation. Reaction conditions: alcohol (1 mmol), H_2_O_2_ (30%, 0.6 mmol) and CS-CDNS-HPA@MIL-101 (60 g) were mixed in H_2_O (7 mL) and then, malononitrile (1.2 mmol) was added and the reaction was stirred at stirred at 55 °C for 1.5 h.

**Table S1.** The effect of solvent on the conversion of the model alcohol oxidation reaction ^a^.

| Entry | Solvent | Conversion (%) | Yield (%) |
| --- | --- | --- | --- |
| 1 | H_2_O | 90 | 90 |
| 2 | CH_3_CN | 81 | 77 |
| 3 | THF | 75 | 70 |
| 4 | EtOH | 90 | 90 |

^a^The reaction was performed using 60 mg CS-CDNS-HPA@MIL-101 and 2 mmol H_2_O_2_ at 55 °C.

**Table S2.** The effect of catalyst on the time, conversion, and yield of product for the oxidation reaction of various alcohols catalyzed by CS-CDNS-HPA@MIL-101 ^a^

| Entry | Substrate | Product | Time (min) | Conversion (%) | Yield (%)^b^ |
| --- | --- | --- | --- | --- | --- |
| 1 |  |  | 75 | 90 | 90 |
| 2 |  |  | 40 | 95 | 95 |
| 3 |  |  | 75 | 90 | 90 |
| 4 |  |  | 75 | 90 | 90 |
| 5 |  |  | 90 | 85 | 85 |
| 6 |  |  | 75 | 95 | 95 |
| 7 |  |  | 90 | 90 | 90 |
| 8 |  |  | 90 | 87 | 87 |
| 9 |  |  | 120 | 75 | 75 |
| 10 |  |  | 120 | 75 | 75 |
| 11 |  |  | 120 | 77 | 77 |

^a^Reaction conditions: alcohol (1 mmol), H_2_O_2_ (2 mmol), CS-CDNS-HPA@MIL-101 60 mg in H_2_O at 55 °C.

^b^Isolated yield

**Table S3.** The effect of catalyst on the time, conversion, and yield of product for the one-pot cascade oxidation-Knoevenagel condensation reaction with various substrates catalyzed by CS-CDNS-HPA@MIL-101 ^a^.

| Entry | Substrate | Product | Time (min)^b^ | Conversion (%) | Yield (%)^b^ |
| --- | --- | --- | --- | --- | --- |
| 1 |  |  | 30 | 90 | 90 |
| 2 |  |  | 30 | 95 | 95 |
| 3 |  |  | 60 | 95 | 95 |
| 4 |  |  | 60 | 92 | 92 |
| 5 |  |  | 60 | 90 | 90 |
| 6 |  |  | 60 | 90 | 90 |
| 7 |  |  | 90 | 90 | 90 |
| 8 |  |  | 60 | 90 | 90 |
| 9 |  |  | 120 | 87 | 87 |
| 10 |  |  | 120 | 80 | 80 |
| 11 |  |  | 120 | 80 | 80 |

^a^Reaction conditions: benzyl alcohol (1 mmol), malononitrile (1 mmol), H_2_O_2_ (2 mmol), CS-CDNS-HPA@MIL-101 (60 mg), 55 °C in water as solvent

^b^Time of Knoevenagel reaction

^c^Isolated yield


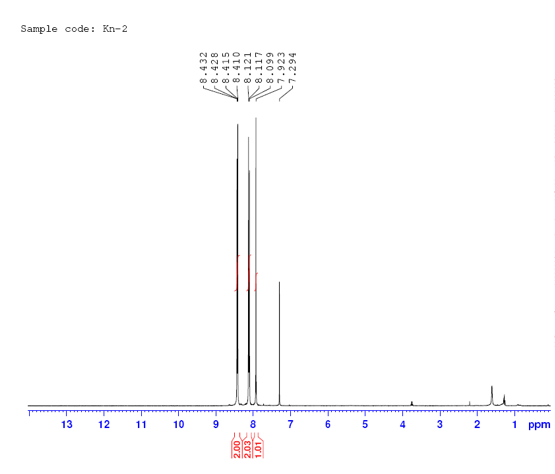


Figure S3. ^1^HNMR spectrum of 2-(4-nitrobenzylidene)malononitrile


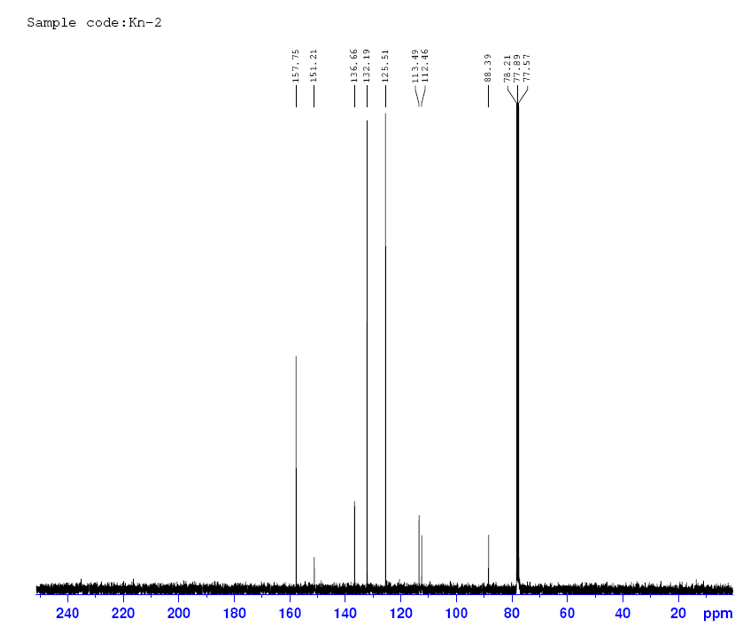


Figure S4. ^13^CNMR spectrum of 2-(4-nitrobenzylidene)malononitrile


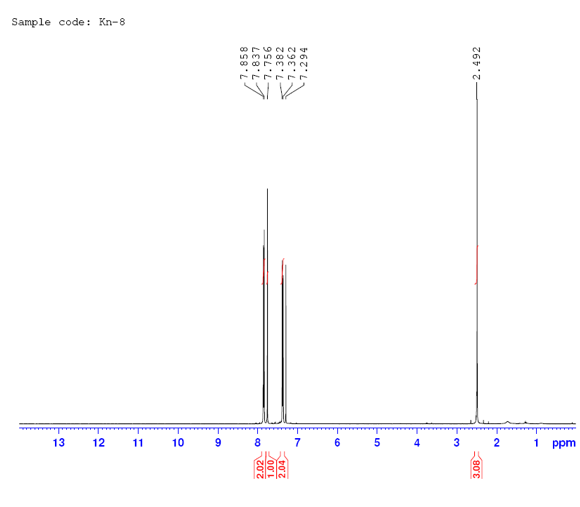


Figure S5. ^1^HNMR spectrum of 2-(4-methylbenzylidene)malononitrile


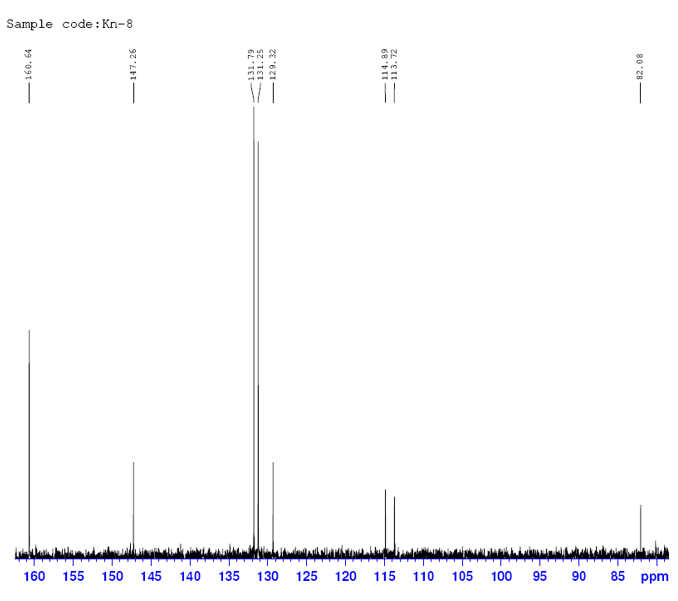


Figure S6. ^13^CNMR spectrum of 2-(4-methylbenzylidene)malononitrile


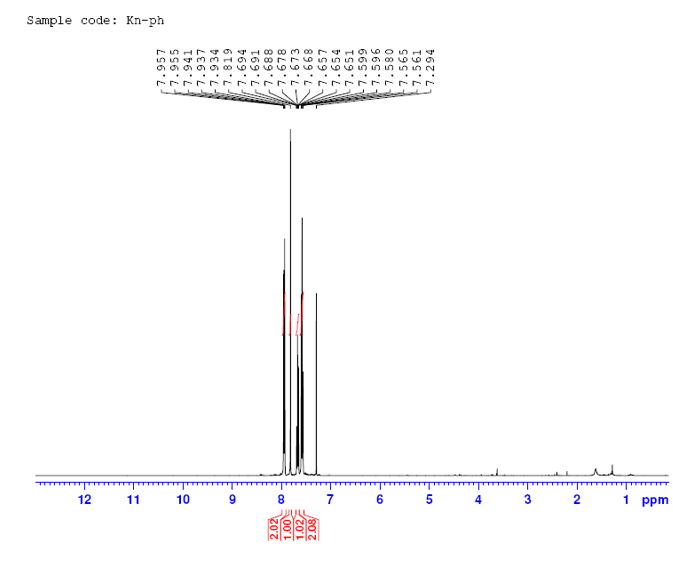


Figure S7. ^1^HNMR spectrum of 2-benzylidenemalononitrile


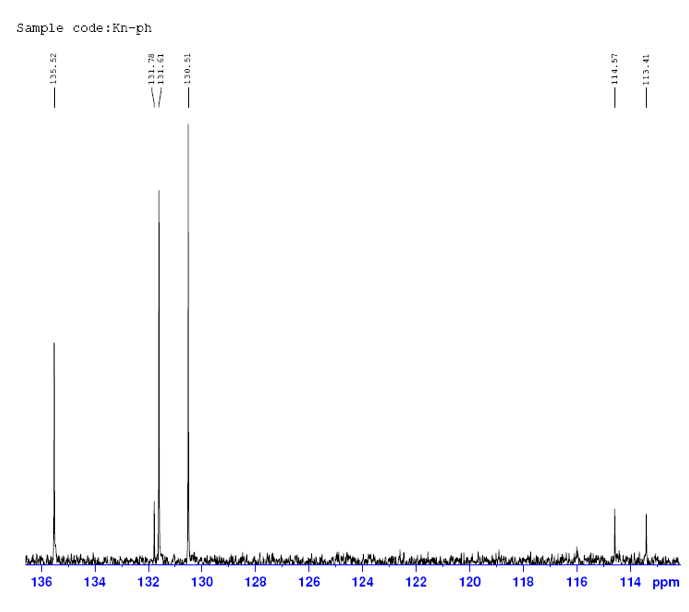


Figure S8. ^13^CNMR spectrum of 2-benzylidenemalononitrile


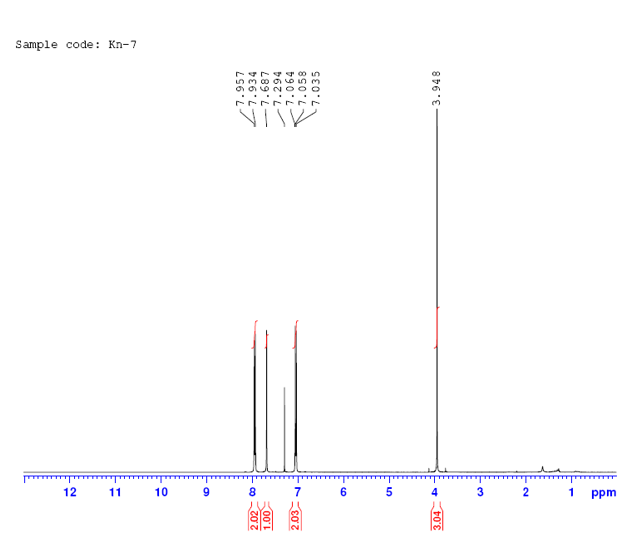


Figure S9. ^1^HNMR spectrum of 2-(4-methoxybenzylidene)malononitrile


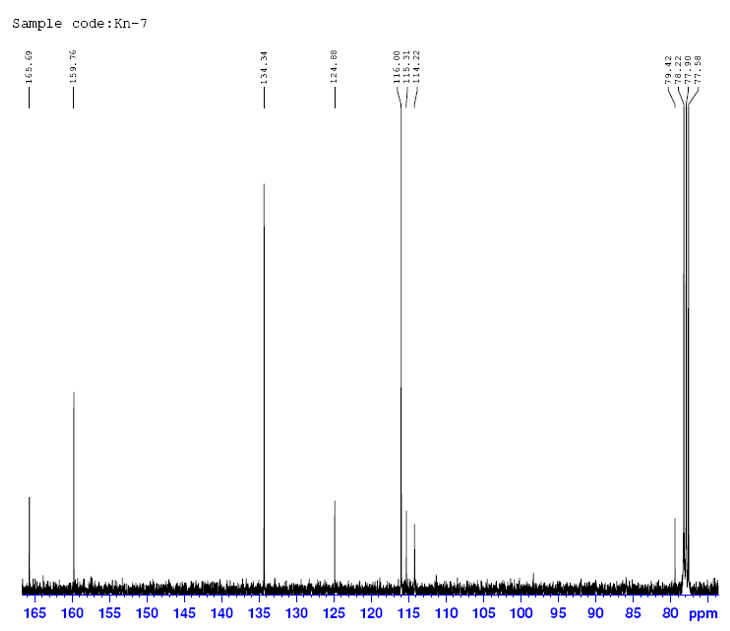


Figure S10. ^13^CNMR spectrum of 2-(4-methoxybenzylidene)malononitrile


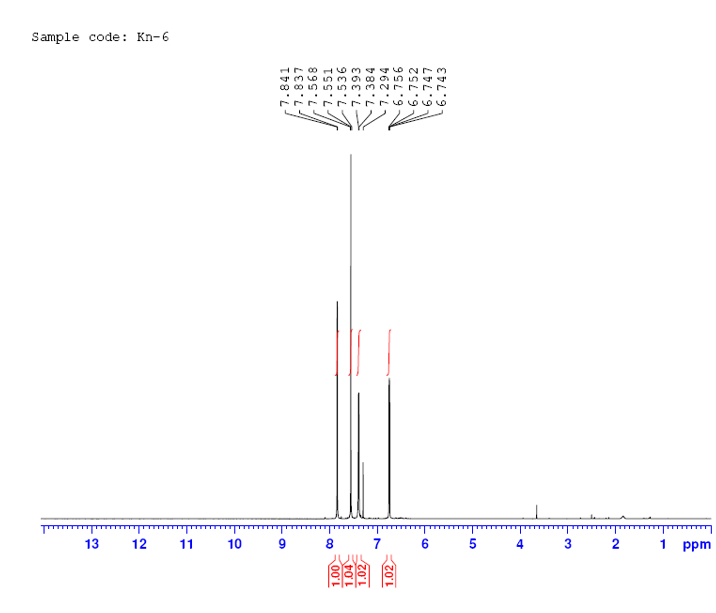


Figure S11. ^1^HNMR spectrum of 2-(furan-2-ylmethylene)malononitrile


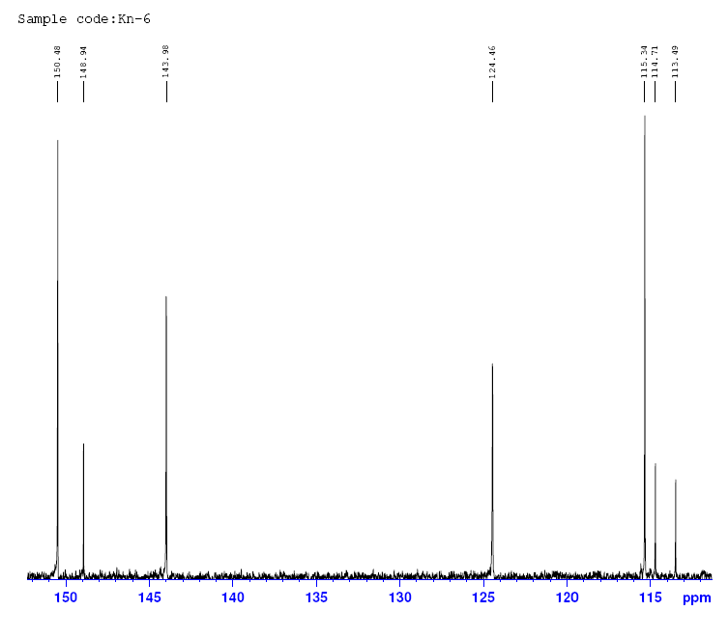


Figure S12. ^13^CNMR spectrum of 2-(furan-2-ylmethylene)malononitrile
